# Commentary on ‘Surgical outcomes and survival following esophagectomy for squamous cell carcinoma with or without liver cirrhosis: retrospective cohort study’

**DOI:** 10.1097/JS9.0000000000001260

**Published:** 2024-03-18

**Authors:** Kexun Li, Xuefeng Leng, Lin Peng

**Affiliations:** Department of Thoracic Surgery, Sichuan Clinical Research Center for Cancer, Sichuan Cancer Hospital & Institute, Sichuan Cancer Center, Affiliated Cancer Hospital of University of Electronic Science and Technology of China (Sichuan Cancer Hospital), Chengdu, People’s Republic of China

*Dear Editor*,

We have carefully read the paper titled ‘Surgical outcomes and survival following esophagectomy for squamous cell carcinoma with or without liver cirrhosis: retrospective cohort study’ by Young Mog Shim *et al*. The authors conducted a rigorous retrospective cohort study, employing both univariate and multivariate analyses to assess overall survival (OS) while accounting for confounding variables. Their investigation sheds light on the influence of liver cirrhosis (LC) on postoperative and long-term outcomes in patients with esophageal squamous cell carcinoma (ESCC)^1^. By comparing 121 ESCC patients with Child–Pugh class A LC to a larger cohort of 2810 ESCC patients without LC, the authors offer a comprehensive analysis. The Model of End-Stage Liver Disease (MELD) score’s association with severe postoperative complications offers valuable insight. They concluded that ESCC patients with Child–Pugh class A LC experience higher rates of postoperative complications, mortality, and lower OS. While the study by Young Mog Shim and colleagues is well-executed and insightful, there are certain aspects that merit further discussion.

Firstly, authors reported that about 20% of patients underwent neoadjuvant therapy; however, the staging section failed to delineate the distinction between the p stage and yp stage, despite the differing prognoses associated with each^2,3^. It was noted that around 8% of the T0 patients in Table 1^1^ were ypT0 patients. Furthermore, the query was raised regarding the presence of patients in Tis stages and whether there were pT1a patients among the 40% of patients classified as T1 in Table 1^1^, a number exceeding that of neoadjuvant patients. Notably, current guidelines recommend endoscopic submucosal dissection (ESD) as the optimal treatment for stages Tis and T1a of cancer^4,5^. The question was posed as to whether the inclusion of patients who should have received ESD impacted the reliability of the study results.

Then, the univariate and multivariate analyses for OS in Table 3^1^ show that the hazard ratio (HR) of pT1 was 0.691 (0.553–0.863) in the univariate analysis and 1.4 (1.087–1.803) in the multivariate analysis. pT1 appears as a protective factor in the univariate analysis but as a harmful factor in the multivariate analysis. Is there any part that requires correction?

In summary, the study by Young Mog Shim and colleagues offers valuable insights into the impact of LC on ESCC patients’ outcomes, it requires further refinement and discussion of the relevant details to improve the comprehensiveness of the conclusions. This will be crucial for advancing clinical practice and optimizing outcomes for patients with these challenging conditions.

## Ethical approval

None.

## Sources of funding

This work was supported by grants from the National Key Research and Development Program (2022YFC2403400), the Department of Science and Technology of Sichuan Province (2023YFS0044, 24GJHZ0166), Sichuan Province Clinical Key Specialty Construction Project.

## Author contribution

All authors: study concept and design, acquisition, analysis, or interpretation of data; K.L.: drafting of the article and statistical analysis; L.P. and X.L.: administrative, technical, or material support and obtained funding; K.L., X.L., and L.P. had full access to all the data in the study and take responsibility for the integrity of the data and the accuracy of the data analysis.

## Conflicts of interest disclosure

The authors declare no conflicts of interest.

## Research registration unique identifying number (UIN)

None.

## Guarantor

Kexun Li and Lin Peng are guarantors.

## Data availability statement

This study does not involve new data, only data from ‘Surgical outcomes and survival following esophagectomy for squamous cell carcinoma with or without liver cirrhosis: retrospective cohort study’.
